# Supplementary figures and images for: Schistosome infection promotes osteoclast-mediated bone loss
Source: PLoS Pathog. 2021 Mar 18;17(3):e1009462. doi: 10.1371/journal.ppat.1009462 (PMC8009420; doi:10.1371/journal.ppat.1009462)

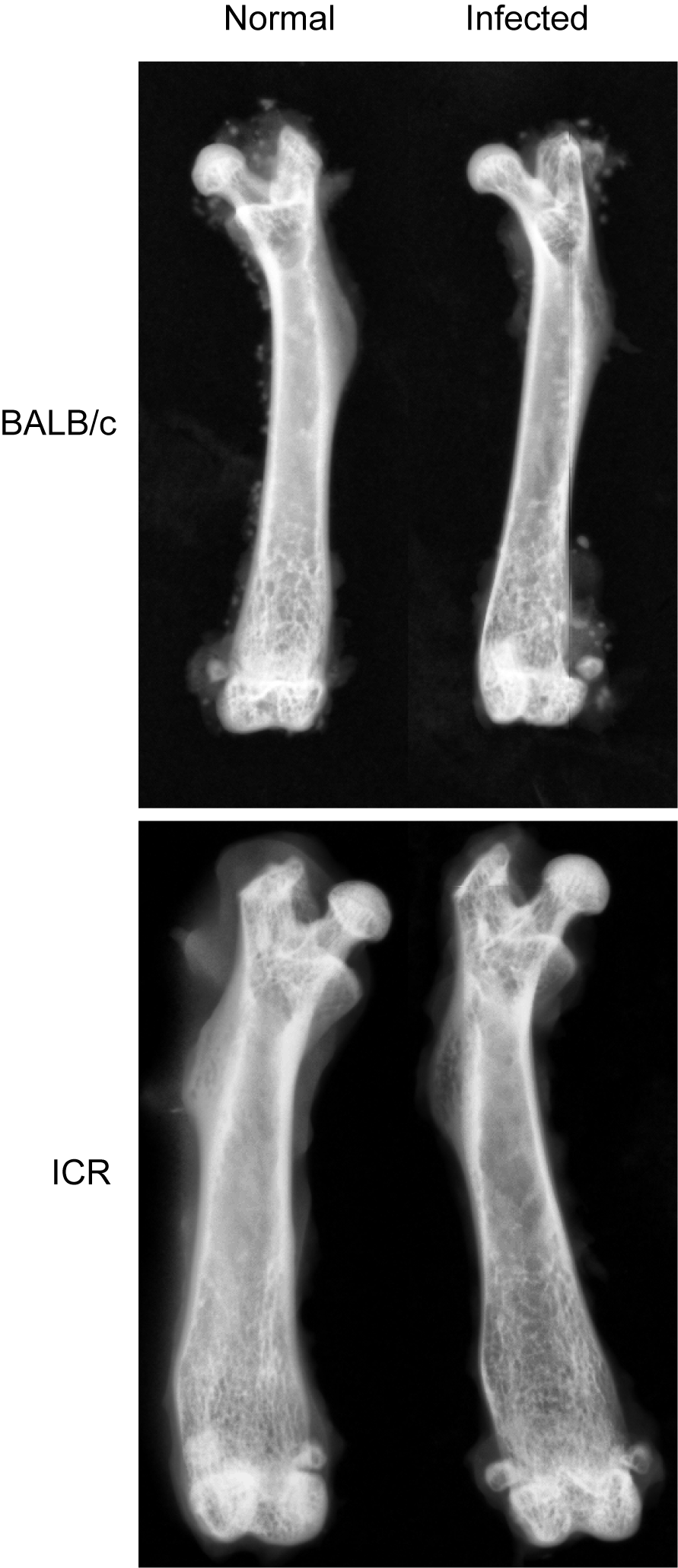

Supplement: S1 Fig — Male BALB/c or ICR mice were infected with 12 cercariae of S. japonicum per mouse. Mice were sacrificed at 13 weeks post-infection. Femurs were isolated from male age-matched normal or S.japonicum-infected mice, and analyzed by X-ray. Representative of X-ray images of femurs. (TIF) [file ppat.1009462.s001.tif]

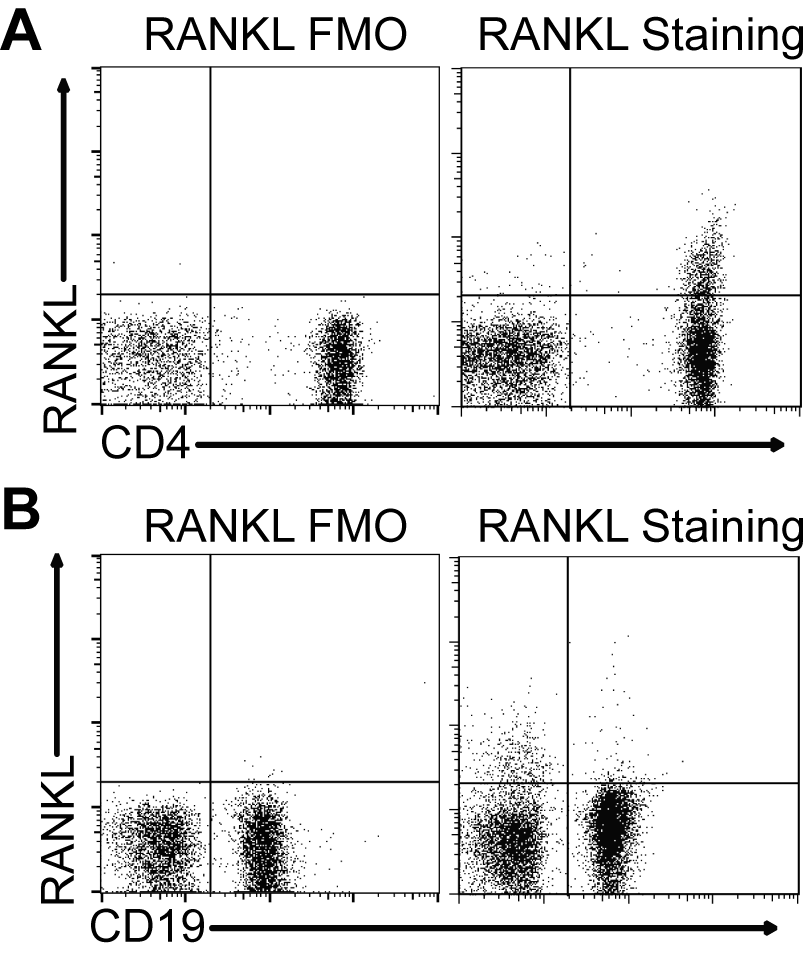

Supplement: S2 Fig — Cells were isolated from the spleen of schistosome-infected mice. (A) Cells were stained with CD3-PerCP-Cy5.5, CD4-APC, and RANKL-PE antibodies. Gated on CD3+ cells. RANKL+CD4+ T cells were analyzed and plotted using Fluorescence Minus One controls (FMO); (B) Cells were stained with CD19-FITC and RANKL-PE antibodies. RANKL+CD19+ B cells were analyzed and plotted using FMO. (TIF) [file ppat.1009462.s002.tif]

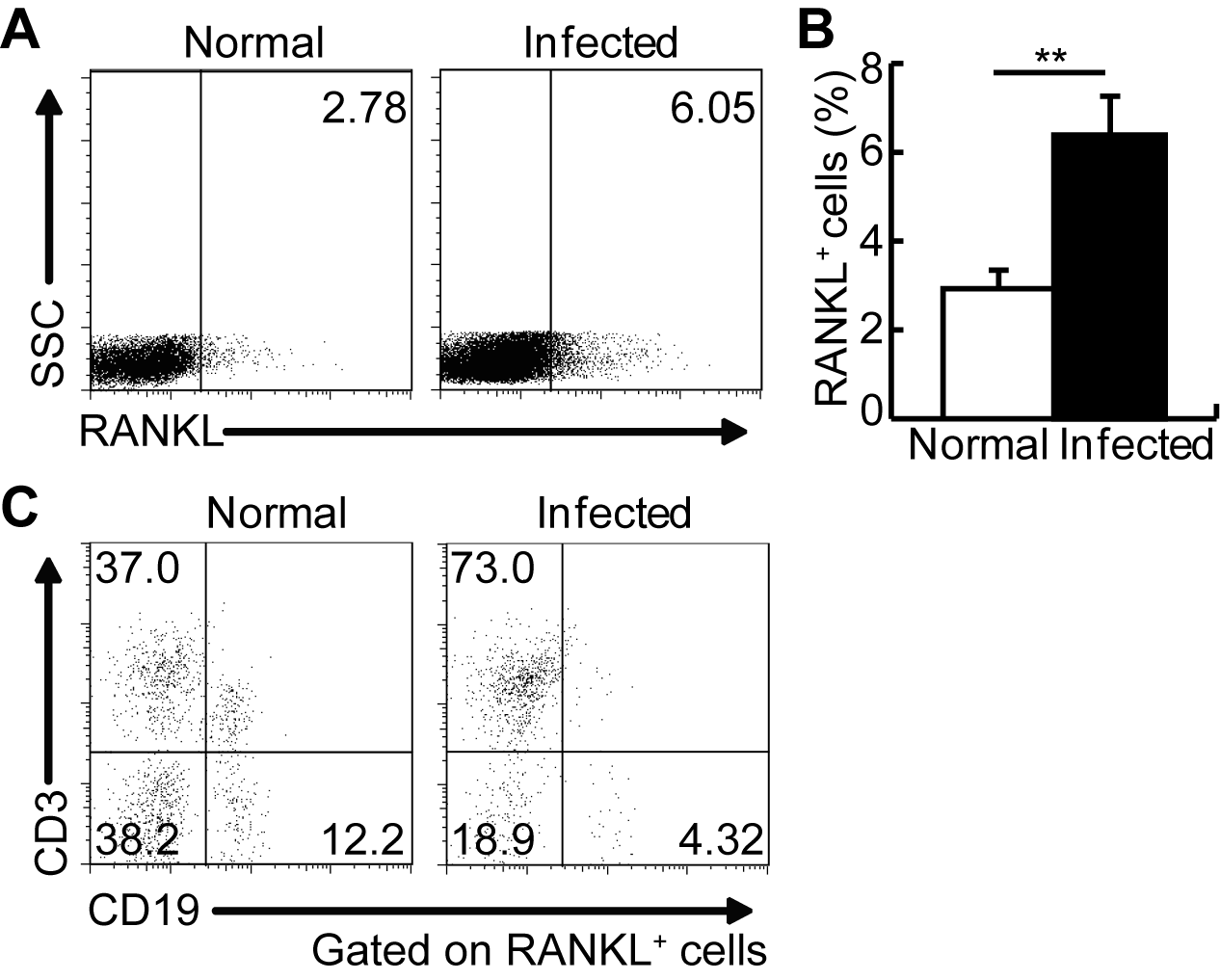

Supplement: S3 Fig — Bone marrow from mice at 13 weeks post-infection or age-matched normal mice was harvested and the cells were surface stained with CD3-PerCP-Cy5.5, CD19-FITC, and RANKL-PE antibodies. (A, B) Representative flow cytometry data plots (A) and statistics (B) show the frequencies of RANKL+ cells in the bone marrow. Data are representative of three independent experiments with 3 mice in each group. **, P<0.01; (C) Representative flow cytometry data plots show the distribution of CD19+ B cells, CD3+ T cells, and CD19-CD3- cells within total RANKL+ cells in bone marrow in infected and normal mice. Gated on RANKL+ cells. Data are representative of three independent experiments with 3 mice in each group. (TIF) [file ppat.1009462.s003.tif]

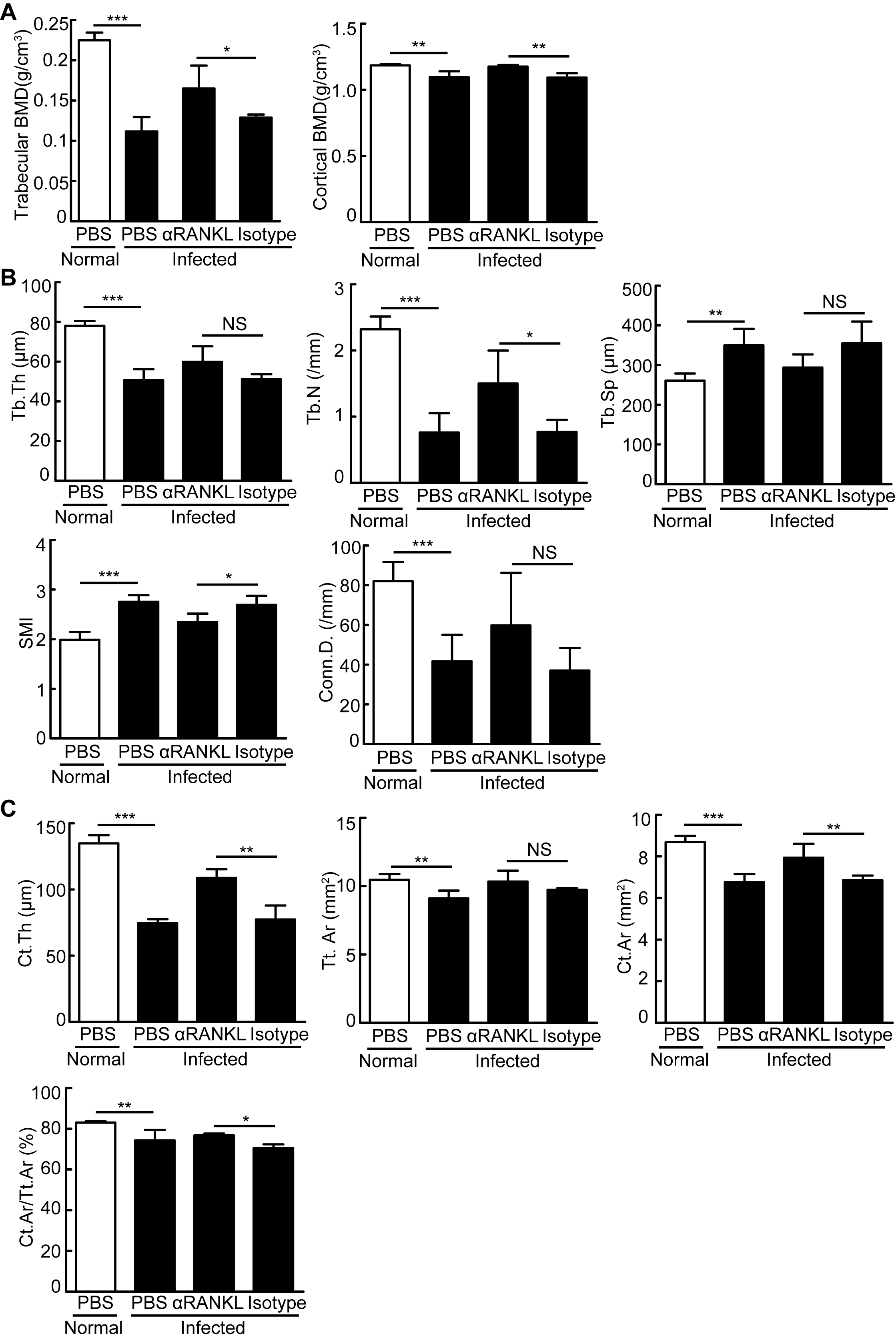

Supplement: S4 Fig — Femurs were isolated from male age-matched normal and S.japonicum-infected mice treated with or without anti-RANKL antibody, and analyzed by microcomputed tomography (μCT) scan (n = 4–5, pool of two independent experiments). (A) Quantitative assessment of trabecular and cortical bone mineral density (BMD); (B) Average quantifications of trabecular thickness (Tb.Th), number (Tb.N.), space (Tb.Sp.), the structure model index (SMI), and connectivity density (Conn.D.); (C) Average quantifications of cortical bone thickness (Ct.Th), total cross-sectional area (Tt.Ar), bone area (Ct.Ar), and cortical bone fraction (Ct.Ar/Tt.Ar). n = 4–5, pool of two independent experiments. Data were expressed as the mean ± SD. *, P<0.05; **, P<0.01; ***, P<0.001, NS indicating not significant. (TIF) [file ppat.1009462.s004.tif]

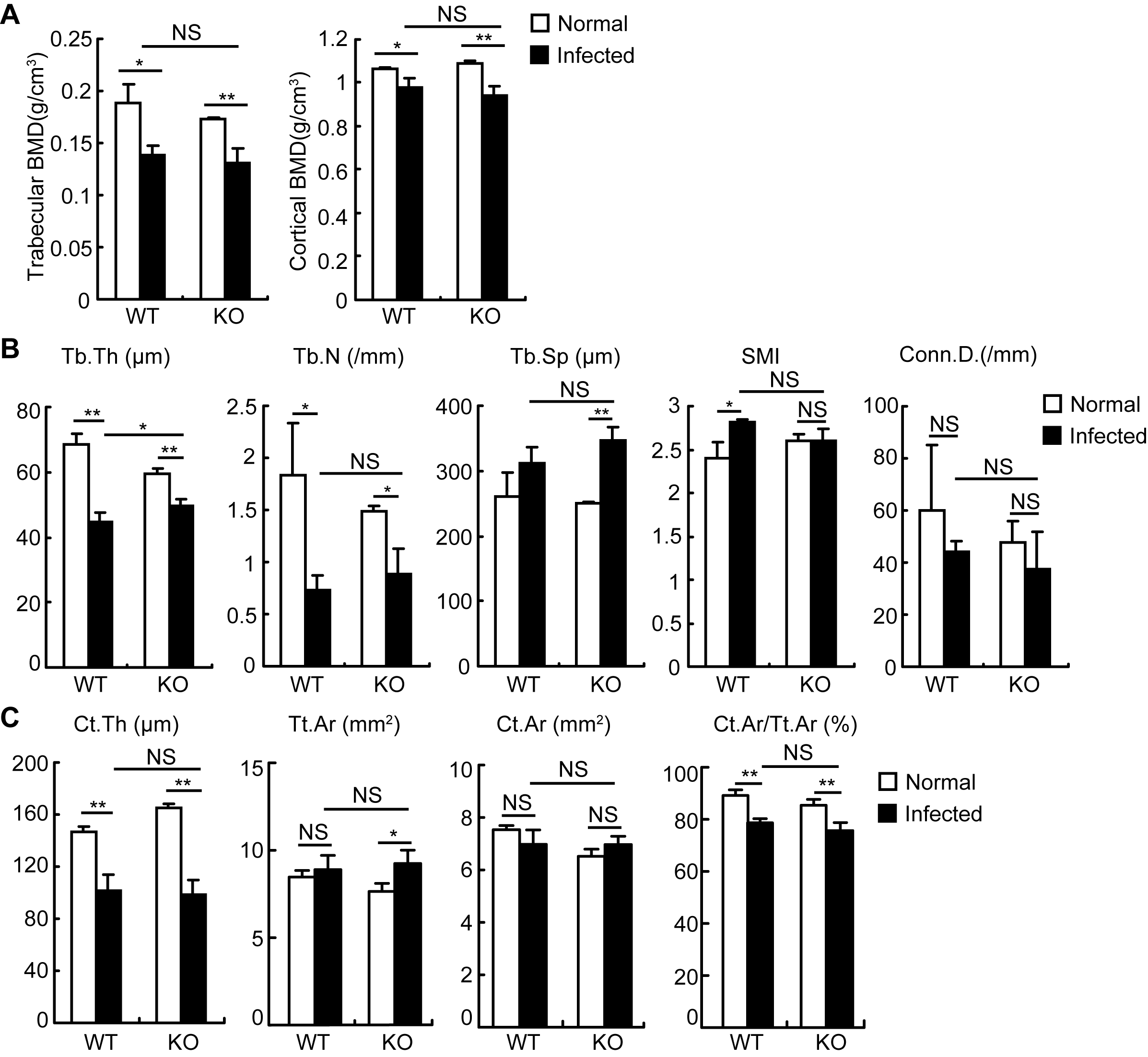

Supplement: S5 Fig — Femurs were isolated from wildtype (WT) or ICOSL knockout (KO) mice infected with or without Schistosoma japonicum, and analyzed by microcomputed tomography (μCT) scan. (A) Quantitative assessment of trabecular and cortical bone mineral density (BMD); (B) Average quantifications of trabecular thickness (Tb.Th), number (Tb.N.), space (Tb.Sp.), the structure model index (SMI), and connectivity density (Conn.D.); (C) Average quantifications of cortical bone thickness (Ct.Th), total cross-sectional area (Tt.Ar), bone area (Ct.Ar), and cortical bone fraction (Ct.Ar/Tt.Ar). Data are representative of two independent experiments with 4 mice in each group. *, P<0.05, **, P<0.01, ***, P<0.001, NS indicating not significant. (TIF) [file ppat.1009462.s005.tif]
