# Supplementary material for: Schistosome infection promotes osteoclast-mediated bone loss
Source: PLoS Pathog. 2021 Mar 18;17(3):e1009462. doi: 10.1371/journal.ppat.1009462 (PMC8009420; doi:10.1371/journal.ppat.1009462)
Supplement: S1 Table — Schistosomiasis patients (n = 238) from a village in Chizhou City, Anhui province, China. The bone mineral density was detected in schistosomiasis patients by the heel ultrasound. The rates of osteoporosis from the healthy controls in Zhejiang province, a previous schistosomiasis endemic area but announced the elimination of this disease in 1992, were published in Chinese academic journal [45]. The rates of osteoporosis or bone loss between schistosomiasis patients and healthy humans were analyzed by Pearson Chi-Square Test. (DOCX) [file ppat.1009462.s006.docx]

**S1 Table.** The rates of bone loss and osteoporosis in schistosomiasis patients and healthy humans

| Age |  | | Healthy Humans | | | | | | | | Schistosomiasis Patients | | | |
| --- | --- | --- | --- | --- | --- | --- | --- | --- | --- | --- | --- | --- | --- | --- |
|  |  | | | Cases(n) | | Normal | | Bone Loss | | Osteoporosis | Cases(n) | Normal | Bone Loss | Osteoporosis |
| 40-49 | | Male | | | 155 | | 126 | | 25 | 4 | 1 | 0 | 1 | 0 |
|  | | Female | | | 199 | | 165 | | 30 | 4 | 5 | 4 | 1 | 0 |
| 50-59 | | Male | | | 178 | | 131 | | 44 | 3 | 13 | 4 | 4 | 5 |
|  | | Female | | | 144 | | 89 | | 53 | 2 | 9 | 3 | 3 | 3 |
| ＞=60 | | Male | | | 112 | | 67 | | 41 | 4 | 55 | 20 | 16 | 19 |
|  | | Female | | | 140 | | 47 | | 86 | 7 | 36 | 4 | 13 | 19 |
| Total | | Male | | | 290 | | 198 | | 85 | 7 | 69 | 24 | 21 | 24 |
|  | | Female | | | 483 | | 301 | | 169 | 13 | 50 | 11 | 17 | 22 |

Schistosomiasis patients (n=238) from a village in Chizhou City, Anhui province, China. The bone mineral density was detected in schistosomiasis patients by the heel ultrasound. The rates of osteoporosis from the healthy controls in Zhejiang province, a previous schistosomiasis endemic area but announced the elimination of this disease in 1992, were published in Chinese academic journal [1]. The rates of osteoporosis or bone loss between schistosomiasis patients and healthy humans were analyzed by Pearson Chi-Square Test.

**Reference**

1. Liu W, Xu CL, Zhu ZQ, Wang W, Han SM, et al. (2006) Bone density of calcaneus of 2769 healthy persons in Zhejiang province. Zhonghua Yi Xue Za Zhi 86: 891-895.
