# Supplementary material for: Schistosome infection promotes osteoclast-mediated bone loss
Source: PLoS Pathog. 2021 Mar 18;17(3):e1009462. doi: 10.1371/journal.ppat.1009462 (PMC8009420; doi:10.1371/journal.ppat.1009462)
Supplement: S1 Text — This file contains detailed materials and methods for S1 Table. (DOC) [file ppat.1009462.s007.doc]

**Supplementary Information**

**Materials and Methods**

**Ethics statement**

Ethical clearance for this study was obtained from the Institutional Review Board of Nanjing Medical University, Nanjing, China (Permit Number: 2014NMUIEC001). The aims and objectives of the study were explained to each participant and written informed consent was obtained. All personal identifiers of the study notes and tapes were kept confidential and destroyed once the study was completed.

**Patients and healthy controls**

The study was conducted on a total of 238 schistosomiasis patients from a village in Chizhou City, Anhui province, China. 238 patients with schistosomiasis japonica by egg detection using the Kato-Katz method with duplicate examination of 3 consecutive stool specimens obtained from each individual. The bone mineral density was detected in schistosomiasis patients by the heel ultrasound. The rates of osteoporosis from the healthy controls in Zhejiang province, a previous schistosomiasis endemic area but announced the elimination of this disease in 1992, were published in Chinese academic journal [45].
