# Supplementary material for: Schistosome infection promotes osteoclast-mediated bone loss
Source: PLoS Pathog. 2021 Mar 18;17(3):e1009462. doi: 10.1371/journal.ppat.1009462 (PMC8009420; doi:10.1371/journal.ppat.1009462)
Supplement: Attachment — Submitted filename: Responses to reviewers.docx [file ppat.1009462.s008.docx]

We appreciate the helpful and constructive comments of the reviewers. We are pleased that all reviewers considered our study to be novel, interesting, and of great benefit to the field. As suggested by the reviewers, we have added the new data and extensively revised our manuscript. The newly acquired results further support the conclusions described in the manuscript.

Our point-by-point responses to the reviewers’ comments are described below:

Reviewer's Responses to Questions

**Part I - Summary**

Please use this section to discuss strengths/weaknesses of study, novelty/significance, general execution and scholarship.

Reviewer #1:

This study investigates the relationships between chronic schistosome infection and bone metabolism in a mouse model. The data suggest schistosome infection induces bone resorption, increased RANKL by follicular helper T cells and diminished OPG. While the studies and outcomes are very interesting, the data is somewhat preliminary and there are numerous concerns regarding the experimental design and its rigor that need to be addressed.

We are pleased that this reviewer considered our findings to be interesting.

Reviewer #2:

Schistosomiasis appears to result in immunological changes that impact bone metabolism. To study this, these authors investigated the relationships between chronic schistosome infection and bone metabolism by using a mouse model of chronic schistosomiasis. Schistosome infection resulted in osteoclast-mediated bone loss, and assays demonstrated activation of NF-κB ligand and decreased level of a molecular, osteoprotegerin. The blockade of NF-κB ligand could prevent the bone loss, which was quite an intriguing result. Follicular helper T (Tfh) cells were identified as a predominant cellular source of NF-κB ligand during schistosome infection. These results might point the way to novel therapeutic interventions against bone loss in schistosome-infected patients.

1. In general, the paper is well written and the conclusions are supported by the data. Appropriate controls and statistical comparisons are provided. One comment is that on page 10 the authors note that many other infections can perturb bone metabolism and growth. This begs the obvious question that the authors should address: namely, how confident can they be that the perturbations are unique to schistosomiasis? Were experiments done with other pathogens to demonstrate that the perturbations in NF-κB ligand and the decreased level of osteoprotegerin were unique to Schistosomiasis or are these noted in other infections?

We are pleased that this reviewer considered our results to be intriguing. Although we haven’t done experiments with other infection by ourselves, other groups have already published their works to show that the increases in the ratio of RANKL to OPG also accelerate the rate of osteoclastic bone resorption in other infectious diseases such as HIV infection and bacterial infection-induced periodontitis [1, 2].

1. Cochran DL (2008) Inflammation and bone loss in periodontal disease. J Periodontol 79: 1569-1576.

2. Vikulina T, Fan X, Yamaguchi M, Roser-Page S, Zayzafoon M, et al. (2010) Alterations in the immuno-skeletal interface drive bone destruction in HIV-1 transgenic rats. Proc Natl Acad Sci U S A 107: 13848-13853.

2. More comment on the precise scope of the skeletal abnormalities in humans would improve the discussion. Are the extensive abnormalities noted in mice also seen in humans? How big of a public health impact does Schistosomiasis have on human bone disease?

As far as we known, no data about the rates of osteoporosis in schistosomiasis-endemic areas has been published so far. As shown in the following table, we compared the rates of osteoporosis in schistosomiasis-endemic areas versus non-endemic (previous endemic) areas. We recruited 238 patients with schistosomiasis from a village in Chizhou City, Anhui province, China, and detected the bone mineral density by the heel ultrasound. The rates of osteoporosis from the healthy controls in Zhejiang province, a previous schistosomiasis endemic area but announced the elimination of this disease in 1992, were published in Chinese academic journal (Liu Wei et al, National Medical Journal of China 2006). Our result indicated that the rates of osteoporosis in schistosomiasis patients were considerably higher than those in healthy controls in previous endemic area (Table S1), while leaves the mechanisms to be unknown and needs investigation in the future. However, these healthy controls are not from the exactly same area as schistosomiasis patients, and it is very difficult to recruit naïve human in schistosomiasis-endemic area.

As suggested by the reviewer, we added more discussion on the skeletal abnormalities in patients with schistosomiasis in Section of Discussion in our revised manuscript on page 11 lines 261-265 as follows: “Indeed, the rates of osteoporosis in schistosomiasis patients were considerably higher than those in healthy controls in non-endemic area. Thus, these results suggest that patients with schistosomiasis, affecting more than 200 million people worldwide, might potentially benefit from coupling bone therapy with anti-schistosome treatment.”

Reviewer #3:

Manuscript PPATHOGENS-D-20-01214, entitled Schistosome Infection Promotes Osteoclast-mediated Bone Loss by Su and Chan proposes that infection with parasitic schistosomes results in osteoclast mediated bone loss by decreasing osteoprotegrin levels potentially contributed to via Tfh production of RANKL. This manuscript proposes that schistosome modulation of the immune response negatively affecting bone density is novel. The data are impressive and add an additional mortality consideration to schistosome infection.

Overall, the potential for this manuscript’s impact is high. However, there are several areas that need to be clarified and amended. In general, the figures should be larger in size. Much of the writing for several of the figures is too small to see and out of focus when enlarged. Also, some of the images should be better labeled. Finally, there are several areas that should be clarified or explained in the document or introduced more clearly.

(Finally, I think this manuscript is on its way to becoming potentially exciting paper. The concept is novel and would be of great benefit to the field. However, again there are several edits that should be made to the document).

We are pleased that this reviewer considered our findings to be novel and of great benefit to the field. As suggested by the reviewer, we have double-checked all figures and increased image size.

**Part II – Major Issues: Key Experiments Required for Acceptance**
Please use this section to detail the key new experiments or modifications of existing experiments that should be absolutely required to validate study conclusions.

Generally, there should be no more than 3 such required experiments or major modifications for a "Major Revision" recommendation. If more than 3 experiments are necessary to validate the study conclusions, then you are encouraged to recommend "Reject".

Reviewer #1:

1. The therapeutic study where RANKL was ablated in the mice to ameliorate bone loss, is poorly explained and the data consists of a single panel of X-ray data. This is not quantitative data and the images are not informative. Full uCT and turnover markers are needed for this study. Irrespective, it’s unclear what this data means and the conclusions of this study are erroneous, and do not prove as claimed, that aberrant RANKL underlies the mechanism. Blocking RANKL would stall bone resorption irrespective of the mechanism by suppressing basal resorption. At the very least an uninfected control group treated with RANKL antibody needs to be performed for comparison. But even with that, interpretation would be difficult. A T cell conditional RANKL KO experiment would be the most convincing, however B cells also appear to make RANKL in this model (the authors dismiss this) and other source such as BMSC and osteocytes that are responsive to IL-17 from Th17, were not investigated as putative sources. The relevant sources of RANKL remain unclear at this time.

As suggested by the reviewer, we have performed bone morphometric analysis of distal femurs from mice treated with or without anti-RANKL by μCT, and added these data into new Figure 3F, Figure 3G, and Figure S4. μCT analysis showed that RANKL blockade ameliorated bone loss in *S. japonicum*-infected mice in our revised manuscript.

The reason why an uninfected control group treated with RANKL blocking antibody was not designed in our study is based on: 1) It is well demonstrated that RANKL deficiency or inhibition suppresses basal bone resorption and consequently results in severe osteopetrosis and stunted growth under physiological conditions [1 2]. 2) Many published works have also supported that the control group of health mice treated with an antibody targeting RANKL signaling was not necessary for investigating the effect of RANKL signaling in pathogenic bone loss [3-6].

We agree with the reviewer that several types of cells, such as B cells and CD4^+^ T cells, are sources of RANKL in schistosome-infected mice. However, it is difficult to determine the relative contribution of these cell types to RANKL production *in vivo* by experiment including conditional RANKL KO in T cells or subtypes, considering the possible compensation from other cells. Our results showed that 60% of RANKL^+^ cells were CD3^+^ T cells in mice 8 or 13 weeks after schistosome infection while only 30% of RANKL^+^ cells were B cells (Figures 4A and 4B). Thus, T cells are likely to be a major contributor to increased RANKL in mice 8 or 13 weeks after schistosome infection. Although IL-17 produced by Th17 cells has a role in promoting BMSC or osteocytes to secrete RANKL, the number of Th17 cells was relative low in schistosome-infected mice (only 0.6% in total CD4^+^ T cells, shown in Fig 5A). Thus, it is plausible that IL-17-mediated RANKL production from other cells may not be a dominant contributor to increased RANKL in schistosome-infected mice. However, the exact contribution of IL-17 to RANKL production in schistosome-infected mice remains to be further investigated. We have added the discussion about these limitations of our study in our revised manuscript on page 12 lines 300-305.

1. Theill LE, Boyle WJ, Penninger JM (2002) RANK-L and RANK: T cells, bone loss, and mammalian evolution. Annu Rev Immunol 20: 795-823.

2. Lezot F, Chesneau J, Navet B, Gobin B, Amiaud J, et al. (2015) Skeletal consequences of RANKL-blocking antibody (IK22-5) injections during growth: mouse strain disparities and synergic effect with zoledronic acid. Bone 73: 51-59.

3. Qian H, Yuan H, Wang J, Du Y, Zhang X, et al. (2014) A monoclonal antibody ameliorates local inflammation and osteoporosis by targeting TNF-alpha and RANKL. Int Immunopharmacol 20: 370-376.

4. Luo J, Yang Z, Ma Y, Yue Z, Lin H, et al. (2016) LGR4 is a receptor for RANKL and negatively regulates osteoclast differentiation and bone resorption. Nat Med 22: 539-546.

5. Zhou L, Hong G, Li S, Liu Q, Song F, et al. (2020) Fangchinoline protects against bone loss in OVX mice via inhibiting osteoclast formation, bone resorption and RANKL-induced signaling. Int J Biol Sci 16: 309-319.

6. Lin X, Han X, Kawai T, Taubman MA (2011) Antibody to receptor activator of NF-kappaB ligand ameliorates T cell-mediated periodontal bone resorption. Infect Immun 79: 911-917.

2. The reporting of methodology and required indices for uCT, does not correspond to the published guidelines (Bouxsein, M. L., et al. (2010). "Guidelines for assessment of bone microstructure in rodents using micro-computed tomography." Journal of Bone and Mineral Research 25(7): 1468-1486). The reported indices and methodology should be revised extensively per these guidelines.

We did follow the guidelines mentioned by this reviewer. However, it is very difficult to cover all indices listed in the guidelines in one study under the limitation of equipment or software. In our study, based on our equipment or software, we followed the guidelines mentioned by this reviewer [1] and chose the following key indices according to substantial previous reports [2, 3]: trabecular bone mineral density (BMD), tissue volume (Tb. TV), bone volume (Tb. Vol), bone volume over tissue volume (Tb. Vol/TV), trabecular thickness (Tb.Th), trabecular number (Tb.N), trabecular space (Tb.Sp), connectivity density (Conn.D.) and structure model index (SMI, an indicator of the trabecular structures); Cortical bone mineral density (BMD), cortical thickness (Co.Th), cortical volume (Co.Vol), cortical bone volume as a fraction of total bone volume (Co. Vol/TV) and total bone volume (Co. TV).

As suggested by the reviewer, we have added the methodology of micro-CT in the Section of Materials and Methods in our revised manuscript on page 15 lines 352-362.

1. Bouxsein ML, Boyd SK, Christiansen BA, Guldberg RE, Jepsen KJ, et al. (2010) Guidelines for assessment of bone microstructure in rodents using micro-computed tomography. J Bone Miner Res 25: 1468-1486.

2. Geng Q, Gao H, Yang R, Guo K, Miao D (2019) Pyrroloquinoline Quinone Prevents Estrogen Deficiency-Induced Osteoporosis by Inhibiting Oxidative Stress and Osteocyte Senescence. Int J Biol Sci 15: 58-68.

3. Li Y, Toraldo G, Li A, Yang X, Zhang H, et al. (2007) B cells and T cells are critical for the preservation of bone homeostasis and attainment of peak bone mass in vivo. Blood 109: 3839-3848.

3. Elevated SMI would not mask the lack of changes in Tb. Sp. as suggested. SMI is a mathematical extrapolation of bone architecture based on a structural model, while Tb. Sp. is an actual measurement. Why Tb. Tp. Does not change is perplexing given both Tb. N. and Tb. Th. Are significantly diminished.

As suggested by the reviewer, we revised our manuscript on page 6 lines 117-120 as follows: “No significant difference in trabecular space (Tb.Sp) was detected, while bone architecture was dramatically altered reflected by an elevated structure model index (SMI) in schistosome-infected mice (Fig 1D).”

These findings are in accordance with the prior reports that T-cell-deficient nude mice and CD40 KO mice displayed a decrease in BV, BV/TV, Tb. Th and Conn., no difference in Tb.Sp, but an elevated SMI [1]. However, it remains unclear why Tb.Sp is not altered while Tb. N. and/or Tb. Th. are significantly diminished in schistosome-infected mice, T-cell-deficient nude mice or CD40 KO mice.

1. Li Y, Toraldo G, Li A, Yang X, Zhang H, et al. (2007) B cells and T cells are critical for the preservation of bone homeostasis and attainment of peak bone mass in vivo. Blood 109: 3839-3848.

4. How was cortical and trabecular BMD calculated by uCT? These is not a traditional uCT indices! TV.D (mg HA per cm3) is the recommended uCT parameter.

The trabecular and cortical bone mineral density (BMD) were calculated automatically with the CT-Analyser (CTAn; Bruker Micro-CT Software).

Following the guidelines mentioned by the reviewer, the minimal set of variables that should be reported for trabecular regions includes BV/TV, Tb.Th, Tb.Sp, and Tb.N because these indices can be found in most publications and also can be compared to some extent with classical histomorphometric variables [1]. All of these recommended indices are presented in our study, and have been able to accurately reflect bone density according to published reports [2, 3], however, TV. D suggested by the reviewer, is not included in the Bruker Micro-CT software we used.

1. Bouxsein ML, Boyd SK, Christiansen BA, Guldberg RE, Jepsen KJ, et al. (2010) Guidelines for assessment of bone microstructure in rodents using micro-computed tomography. J Bone Miner Res 25: 1468-1486.

2. Geng Q, Gao H, Yang R, Guo K, Miao D (2019) Pyrroloquinoline Quinone Prevents Estrogen Deficiency-Induced Osteoporosis by Inhibiting Oxidative Stress and Osteocyte Senescence. Int J Biol Sci 15: 58-68.

3. Li Y, Toraldo G, Li A, Yang X, Zhang H, et al. (2007) B cells and T cells are critical for the preservation of bone homeostasis and attainment of peak bone mass in vivo. Blood 109: 3839-3848.

5. There are many required cortical indices that are missing and should to be presented, especially given the image seems to suggest significant decline in cortical bone mass.

According to the guidelines mentioned by the reviewer and previous published reports [1, 2], we have analyzed cortical bone volume as a fraction of total bone volume (Co. Vol/TV) and total bone volume (Co. TV) besides cortical bone mineral density (BMD), cortical thickness (Co. Th), and cortical volume (Co. Vol).

1. Bouxsein ML, Boyd SK, Christiansen BA, Guldberg RE, Jepsen KJ, et al. (2010) Guidelines for assessment of bone microstructure in rodents using micro-computed tomography. J Bone Miner Res 25: 1468-1486.

2. Luo J, Yang Z, Ma Y, Yue Z, Lin H, et al. (2016) LGR4 is a receptor for RANKL and negatively regulates osteoclast differentiation and bone resorption. Nat Med 22: 539-546.

6. Analysis of the axial skeleton should also be performed.

The reason why the femurs rather than axial skeleton were used to investigate the bone loss in our study is based on: 1) The femurs are extensively employed to exclusively investigate the bone loss in many publications [1-4], because of the accuracy and reproducibility of the results from the femurs according to the guidelines mentioned by the reviewer [5]. 2) The femurs can be used to evaluate both trabecular and cortical parameters according to the guidelines. 3) Although the axial skeleton also can be used for cortical bone measurements, it is traditionally used for trabecular bone measurements according to the guidelines.

1. Geng Q, Gao H, Yang R, Guo K, Miao D (2019) Pyrroloquinoline Quinone Prevents Estrogen Deficiency-Induced Osteoporosis by Inhibiting Oxidative Stress and Osteocyte Senescence. Int J Biol Sci 15: 58-68.

2. Li Y, Toraldo G, Li A, Yang X, Zhang H, et al. (2007) B cells and T cells are critical for the preservation of bone homeostasis and attainment of peak bone mass in vivo. Blood 109: 3839-3848.

3. Vikulina T, Fan X, Yamaguchi M, Roser-Page S, Zayzafoon M, et al. (2010) Alterations in the immuno-skeletal interface drive bone destruction in HIV-1 transgenic rats. Proc Natl Acad Sci U S A 107: 13848-13853.

4. Lee MSJ, Maruyama K, Fujita Y, Konishi A, Lelliott PM, et al. (2017) Plasmodium products persist in the bone marrow and promote chronic bone loss. Sci Immunol 2.

5. Bouxsein ML, Boyd SK, Christiansen BA, Guldberg RE, Jepsen KJ, et al. (2010) Guidelines for assessment of bone microstructure in rodents using micro-computed tomography. J Bone Miner Res 25: 1468-1486.

7. Figure 4 shows that both CD4 and CD19 lymphocytes make RANKL during infection, however the authors conclude that only T cells contribute to bone resorption. This is surprising. The data also needs to be normalized for total cell number as % is very misleading. The number of total B cells in the spleen and bone marrow is significantly higher than that of T cells, and hence even a small change in B cell RANKL may considerably outweigh a relatively larger increase in T cell RANKL. More detailed studies would be needed to ascertain the relevant sources of RANKL. Why were CD8 T cells excluded from the study, they are also known to make RANKL.

To rule out the possibility that the ratios is misleading, we analyzed the distributions, which reflect the comparison of the cell numbers, of total RANKL-producing cells, and found that 60% and 70% of total RANKL-producing cells were T cells in the spleen and bone marrow from infected mice, respectively (Figure 4A-4B, Figure S3C). However, only 30% of total RANKL-producing cells were B cells. Thus, although both B cells and T cells are sources of RANKL in schistosome-infected mice, T cells rather than B cells, are likely to be a major contributor to increased RANKL during schistosome infection.

In addition, 90% of RANKL^+^ T cells were CD4^+^ T cells, while 10% of RANKL^+^ T cells were CD8^+^ T cells (CD3^+^CD4^-^ cells; shown in Figure 4A-4B) in mice 8 or 13 weeks after schistosome infection. Thus, most of RANKL^+^ T cells were CD4^+^ T cells during schistosome infection.

8. Th17 T cells are not only producers of RANKL, they are inducer of RANKL from other cells including osteoblast lineage cells, which were not examined.

We agree with the reviewer that Th17 cells are producer and inducer of RANKL. The number of Th17 cells was very low in schistosome-infected mice (only 0.6% in total CD4^+^ T cells, shown in Fig 5A). The exact contribution of Th17 to RANKL production in schistosome-infected mice remains to be further investigated. We have added the discussion about these limitations in our revised manuscript on page 12 lines 300-305.

9. Figure 5. A full uCT analysis of cortical and trabecular bone and bone turnover markers are needed to substantiate the conclusion that Tfh mediates the bone loss in infected mice. The changes in trabecular BMD (whatever that represents) are less than half of those shown for WT mice. Thus, a partial role for Tfh only may be indicated. That is responsible for the rest of the activity?

As suggested by the reviewer, we have performed bone morphometric analysis of distal femurs from schistosome-infected ICOSL KO mice by μCT, and added these data into new Figures 5H, 5I, and Figure S5 in our revised manuscript.

The μCT analysis of distal femurs showed that Tfh cell deficiency alone was difficult to reverse schistosome infection-induced bone loss, reflected by no significant differences in the indices for uCT between schistosome-infected KO mice and schistosome-infected WT mice (new Fig 5H, 5I, and Fig S5). Thus, our results indicated that Tfh cells played an important role of contributor, which may be sufficient but not necessary, in schistosome infection-induced bone loss.

10. Throughout, the X ray images are not quantitative and are not convincing of an effect.

As suggested by the reviewer, we have performed bone morphometric analysis of distal femurs from mice treated with or without anti-RANKL, or from schistosome-infected ICOSL KO mice by μCT, and added these data into new Figure 3F, 3G, Figure S4, Figure 5H, 5I, and Figure S5 in our revised manuscript.

Reviewer #2: No issues.

Reviewer #3:

1. Many of the figures and their description are too small to read and out of focus when expanded. This should be remedied.

As suggested by the reviewer, we have increased image size.

2. Figure 2B should be better described and clarified so that the reader knows what to look for in the figure (green or purple?). The description in the results as well as the figure legend are somewhat uninformative. In addition, in contrast to the proposed result, that by TRAP staining infected mice had more osteoclasts and larger osteoclast-covered surface, the TRAP staining in Figure 2B appears to show the opposite result relative to the graph on Figure 1C.

We are sorry for omitting the details. In our revised manuscript, we have included more details in the results and figure legends.

In Figures 2B and 2C, the data showed that the ratio of osteoclast surface (purple in figure 2B) to bone surface was significantly increased in schistosome-infected mice. As shown in Figure 1C, trabecular and cortical bone mineral density were significantly decreased in schistosome-infected mice. It is reasonable that the increased osteoclasts resulted in a decrease in trabecular and cortical bone mineral density.

3. P6L22-P7L2-The results here are dependent on Fig 2B which is not convincing as described above. (“Our aforementioned results showed an increase in osteoclast generation in *S.japonicum*-infected mice relative to normal mice, suggesting an increased osteoclast precursor (OCP) formation, elevated the productions of factors involved in the differentiation of OCPs into OCs, or both.”). The observation does not measure for osteoclast generation, it just take a steady state number at one time frame. It is possible that osteoclasts could have increased or decreased viability/longevity in infected mice.

We agree with this reviewer that we may have over-interpreted the data. We have revised the description and interpretation of the data in our revised manuscript to make them more appropriate as follows: “Our aforementioned results showed the increases in bone resorption and osteoclast-covered surface in *S.japonicum*-infected mice relative to normal mice (Fig 2A-2C). We next wondered whether the aberrant osteoclast generation was involved in the increased bone resorption during schistosome infection. Thus, we firstly analyzed the absolute number of OCPs (CD3^-^B220^-^NK1.1^-^CD11b^-/low^CD115^+^CD117^+^ cells) in the bone marrow (BM) of normal and infected mice.”

4. Figure 3E line description needs more information that RANKL blockade ameliorated bone loss… P11L10-12. In addition, Figure 3E is not described in the Figure legend making it uninterpretable. Also, Figure 3E should have an image with greater resolution. Based on the current resolution, the addition of the RANKL blockade is not overly convincing. Greater resolution may help to support the author’s conclusion.

We have added the legend of Figure 3E and the details about Figure 3E in the Section of Result in our manuscript.

We agree with the review that the X-ray images are not quantitative and are not overly convincing. However, the X-ray image we used is already the highest resolution that the machine can provide. To more clearly determine the effect of RANKL blockade on schistosome infection-induced bone loss, we have performed bone morphometric analysis of distal femurs from mice treated with or without anti-RANKL by μCT, and added these data into new Figures 3F, 3G and Figure S4 in our revised manuscript. These data further support our conclusion that RANKL blockade ameliorated schistosome infection-induced bone loss in mice.

5. Figure 4 and the writing is too small and too busy to see. It is also out of focus when expanded. It should be remade for greater readability. Figure 4B is too small to evaluate.

As suggested by the reviewer, we have double-checked all figures and increased image size.

6. Is there a negative control experiment using uninfected mice for Figure 5A and 5B?

Uninfected mice were not used because the percentages and numbers of Th1, Th2, Th17, and Tfh cells were very low or even undetectable in uninfected mice. Thus, it is very difficult to further investigate the distributions of their RANKL^+^ subpopulations in uninfected mice.

7. The justification for only using male mice but not female mice is not clear.

Estrogen plays a fundamental role in skeletal growth and homeostasis, and is used as pharmacological agent to prevent postmenopausal bone loss [1]. As we known, estrogens are a group of sex steroid hormones predominantly found in females. To rule out the possibility that estrogen changes are induced by inflammatory cytokines such as TGF-β effect on schistosome infection-induced bone loss [2], we employed male mice to study the roles of RANKL in bone loss during schistosome infection. However, whether estrogen changes are also involved in schistosome infection-induced bone loss, particularly in female mice, needs to be further investigated.

1. Weitzmann MN, Pacifici R (2006) Estrogen deficiency and bone loss: an inflammatory tale. J Clin Invest 116: 1186-1194.

2. Liu ML, Wang H, Wang ZR, Zhang YF, Chen YQ, et al. (2013) TGF-beta1 regulation of estrogen production in mature rat Leydig cells. PLoS One 8: e60197.

**Part III – Minor Issues: Editorial and Data Presentation Modifications**
Please use this section for editorial suggestions as well as relatively minor modifications of existing data that would enhance clarity.

Reviewer #1:

1. A lot of grammatical errors. Recommend a native English speaker review the manuscript, or Grammar software beyond Word should be utilized.

We would like to thank the reviewer for pointing out our grammatical mistake, and we have carefully edited the manuscript.

2. Please check spelling of “Infected” in Figure 2A.

As suggested by the reviewer, we have double-checked all figures in our revised manuscript.

3. It is suggested that anti-schistosomal drug treatment coupled with Denosumab may be beneficial in improving bone health. Any antiresorptive drug would likely be effective in improving bone mass. There does not seem to be a specific indication for use of Denosumab simply because RANKL is increased. As schistosome infection occurs largely in resource-limiting settings, Denosumab with its high cost would not seem to be a practical option compared to a bisphosphonate which would have similar effect. Furthermore, if schistosomes are eradicated pharmacologically as suggested, would this not alone prevent further bone loss? In which case Denosumab would likely no longer be necessary.

We agree with the reviewer that Denosumab would not seem to be a practical option due to its high cost. Although we can’t rule out the possibility that the limited resource was involved in schistosome infection-induced bone loss in this study, our results from before and after intervention of RANKL are still enough to demonstrate that the increased RANKL contributed to pathogenic bone loss during schistosome infection. As suggested by the reviewer, we have edited the manuscript to make it more practical and reasonable on page 3 lines 48-50, and page 13 lines 320-321.

Mice were treated with Praziquantel (PZQ) at week 8 after infection, and sacrificed 5 weeks after PZQ treatment. Our preliminary results showed that anti-schistosome treatment alone did not dramatically ameliorate bone loss in schistosome-infected mice. Thus, anti-schistosome treatment (Praziquantel) coupled with bone therapy (Denosumab or bisphosphonate) may be more beneficial in improving bone health in schistosome-infected individuals.

4. Page 11 line 17: however, no evidence shows that schistosomes or schistosome-derived antigens have demonstrable effect on osteocytes. The meaning or relevance of this statement is unclear.

As suggested by the reviewer, we have removed this sentence in the Section of Discussion in our revised manuscript.

5. Page 13, line 18: The contention that reference 42 documents RANKL expression by Tregs is not valid. In fact, this interesting study shows that a sub-set of Tregs may transdifferentiate into Th17 cells that secrete RANKL. The Tregs themselves are not generally considered a source of RANKL, and if anything, are anti-osteoclastogenic.

We would like to thank the reviewer for pointing out our weakness. As suggested by the reviewer, we have removed this reference and carefully edited the manuscript to make it more concise as follows: “Although Tfh cells deficiency alone reduced significantly the frequency of RANKL-producing cells in CD4^+^ T cells, it only decreased slightly the percentages of total RANKL-producing cells in total lymphocytes, which suggests that Tfh cells were identified as a predominant subset of RANKL-producing CD4^+^ T cells, however, other RANKL-producing cells could serve a complementary role.”

6. Immunohistochemical studies/ How was the osteoclast quantifications performed? No methodology is presented.

As suggested by the reviewer, we have added this information in the Section of Materials and Methods in our revised manuscript as follows: “Static histomorphometric parameters were measured in sections stained for TRAP using Image-Pro Plus software. The following primary parameters were determined in five fields of view (×200) in one section per mouse: OC surface area and bone surface area. The values obtained from these 5 fields were then averaged and used as the value for that particular animal. From the primary data, the OC surface per bone surface area (Oc.S/BS) was calculated.”

7. Flow methods. No mention of FMO and isotype controls for gating.

We have added this information in flow methods as follows: “All flow cytometry results were analyzed and plotted using Fluorescence Minus One controls (FMO).”

Reviewer #2: No concerns.

Reviewer #3:

1. Page number should be continuous and not restart during every page. This makes reference to pages and page number more challenging.

Page number is set to be continuous in our revised manuscript.

2. The figures should be consistent in representation. In some figures “Infected” and “Normal” are capitalized is capitalized and in other cases it is in lowercase.

As suggested by the reviewer, we have double-checked all figures.

3. Figure 2 A, C, E. Spelling errors (“infectied” should be infected)

We would like to thank the reviewer for pointing out these misspelling, and we have corrected them in Figure 2.

4. RANKL Block should be defined in the manuscript as RANKL blocking antibody.

As suggested by the reviewer, we have defined “RANKL blockade” in our revised manuscript as follows: “The blockade of RANKL by anti-RANKL antibody could prevent the bone loss, indicating that RANKL contributes to the pathological bone resorption in the context of schistosome infection”.

5. On P4, L21, 22 a reference is needed Is this specific to *S. japonicum* or is this for all schistosome species?

As suggested by the reviewers, we have added references in our revised manuscript as follows: “In fact, an increase in the ratio of RANKL to OPG accelerates the rate of osteoclastic bone resorption in various infectious diseases such as HIV infection and bacterial infection-induced periodontitis [1, 2]. However, it is unknown whether the ratio of RANKL to OPG is increased in human or animals infected with *S. japonicum* or other schistosome species.”

1. Cochran DL (2008) Inflammation and bone loss in periodontal disease. J Periodontol 79: 1569-1576.

2. Vikulina T, Fan X, Yamaguchi M, Roser-Page S, Zayzafoon M, et al. (2010) Alterations in the immuno-skeletal interface drive bone destruction in HIV-1 transgenic rats. Proc Natl Acad Sci U S A 107: 13848-13853.

6. P6L7-9 This sentence is unclear.

As suggested by the reviewer, we have revised our manuscript as follows: “No significant difference in trabecular space (Tb.Sp) was detected, while bone architecture was dramatically altered reflected by an elevated structure model index (SMI) in schistosome-infected mice (Fig 1D).” on page 6 lines 117-120.

7. Figure 1E should be highlighted in the Figure Legends. Figure 1E is also not discussed. The use of bone structures is new in the schistosome field. Consequently, the authors might consider describing the anatomy and significance more effectively.

We have added the legend of Figure 1E and the detailed description about the anatomy in Section of Result in our revised manuscript on page 6 lines 110-120 as follows: “X-radiographic analysis of the femurs showed that mice in the chronic phase of the infection (since 11–13 weeks post-infection) had severe osteoporosis accompanied by marked decrease in trabecular bone mineral density (Fig 1A). Microcomputed tomography (μCT) analysis of distal femurs showed that schistosome-infected mice had significantly reduced trabecular bone volume and number (Fig 1B). Bone morphometric analysis of distal femurs by μCT showed that trabecular and cortical bone mineral density (BMD, Fig 1C), trabecular bone volume (Tb. Vol), trabecular bone volume/tissue volume (Tb. Vol/TV), trabecular thickness (Tb.Th), number (Tb.N.), and connectivity density (Conn.D., Fig 1D), as well as cortical bone thickness (Co.Th), volume (Co.Vol), and cortical bone volume/tissue volume (Co. Vol/TV, Fig 1E) were significantly decreased in schistosome-infected mice. However, no significant difference in trabecular or cortical tissue volume (Tb. TV or Co. TV) or trabecular space (Tb.Sp) was detected, while bone architecture was dramatically altered reflected by an elevated structure model index (SMI) in schistosome-infected mice (Fig 1D and 1E). ”

8. Correct spelling errors on P8L9.

We would like to thank the reviewer for pointing out this misspelling, and we have corrected it in our revised manuscript.
