# Supplementary material for: Schistosome infection promotes osteoclast-mediated bone loss
Source: PLoS Pathog. 2021 Mar 18;17(3):e1009462. doi: 10.1371/journal.ppat.1009462 (PMC8009420; doi:10.1371/journal.ppat.1009462)
Supplement: Attachment — Submitted filename: Responses to reviewers.docx [file ppat.1009462.s009.docx]

We appreciate the helpful and constructive comments of the reviewers and the members of the editorial board. We are pleased that the reviewers considered our study to be an important topic and that the editors are likely to accept our manuscript for publication. As suggested by the reviewers, we have reanalyzed the data of μCT and revised our manuscript to interpret the data more appropriately.

Our point-by-point responses to the reviewers’ comments are described below:

Reviewer's Responses to Questions

**Part I - Summary**

Please use this section to discuss strengths/weaknesses of study, novelty/significance, general execution and scholarship.

Reviewer #1:

The authors have addressed in part my concerns, and the manuscript is improved. However, there are still some relatively simple to fix issues that were not effectively addressed and need to be thought through more carefully and fixed. The interest and novelty of the study remains a strength.

As suggested by the reviewers, we have reanalyzed the data of μCT and revised our manuscript to interpret the data more appropriately.

Reviewer #3:

I am satisfied with the modifications and responses and changes made by the authors in response to the reviewers' comments. They have addressed my concerns satisfactorily.

We are pleased that this reviewer is satisfied with our modifications and responses.

**Part II – Major Issues: Key Experiments Required for Acceptance**
Please use this section to detail the key new experiments or modifications of existing experiments that should be absolutely required to validate study conclusions.

Generally, there should be no more than 3 such required experiments or major modifications for a "Major Revision" recommendation. If more than 3 experiments are necessary to validate the study conclusions, then you are encouraged to recommend "Reject".

Reviewer #1: N/A

Reviewer #3: None

**Part III – Minor Issues: Editorial and Data Presentation Modifications**
Please use this section for editorial suggestions as well as relatively minor modifications of existing data that would enhance clarity.

Reviewer #1:

1. The authors have failed to address the previous concerns regarding the role of B cell RANKL production in this model. B cells are still being dismissed based on the fact that a higher percentage of T cells (60%) express RANKL. This is an incorrect comparison because, as previously explained, the total number of B cells are dramatically higher than CD4 T cells, especially in the bone marrow where bone resorption occurs and where B cell subpopulations may represent up to 25% of the total cell population. By comparison CD4 T cells normally represent only 2-3% of total bone marrow. Similar, in spleen, B cells are 50% of total cells while CD4 represent only about 7%. Consequently, even if only half as many B cells are making RANKL (30% is reported here), because there are 5X as many B cells in total, their contribution may be very significant and outweigh that of T cells. Without further data as to the relative contributions of B and T cells, the authors need to stop claiming that T cells, and in particular CD4 T cells, are the major drivers of bone resorption and make allowance for a role of both T and B cells, and potentially a greater role of B cells given their relatively larger numbers.

We agree with this reviewer that we may have over-interpreted the data. As suggested by the reviewer, we have revised the manuscript to interpret the data more appropriately.

In section of Abstract, we replaced “Meanwhile, follicular helper T (Tfh) cells were identified as a predominant cellular source of RANKL during schistosome infection.” with “Meanwhile, both B cells and CD4^+^ T cells, particularly follicular helper T (Tfh) cell subset, were the important cellular sources of RANKL during schistosome infection.” in our revised manuscript on page 2 lines 31-34.

In section of Author Summary, “Furthermore, our results indicated that RANKL^+^CD4^+^ T cells, in the peripheral lymphoid tissues are likely to be an important contributor to RANKL-mediated bone loss through releasing soluble RANKL” was replaced with “Furthermore, our results indicated that both B cells and CD4^+^ T cells, particularly Tfh cell subset, in the peripheral lymphoid tissues are likely to be the important contributors to RANKL-mediated bone loss through releasing soluble RANKL” in our revised manuscript on page 3 lines 44-47.

In section of Introduction, “Here, we found that RANKL promoted schistosome-induced bone loss, and identified Tfh cells as a major contributor to an increased level of RANKL during schistosome infection.” was revised to “Here, we found that the increased RANKL was associated with schistosome infection-induced bone loss, and showed that both B cells and CD4^+^ T cells, particularly Tfh cell subset, were the important contributors to an increased level of RANKL during schistosome infection.” in our revised manuscript on page 5 lines 77-80.

In section of Results, we replaced “CD4^+^ T cells contribute to the increased level of RANKL during schistosome infection” with “Both B cells and CD4^+^ T cells contribute to the increased level of RANKL during schistosome infection” in our revised manuscript on page 8 lines 138-139.

In section of Discussion, we replaced “Tfh cells, a dominant subset of RANKL-producing CD4^+^ T cells, are involved in schistosome infection-induced bone loss *in vivo*.” with “both B cells and CD4^+^ T cells, particularly Tfh cells, a dominant subset of RANKL-producing CD4^+^ T cells, are involved in schistosome infection-induced bone loss *in vivo*.” in our revised manuscript on page 14 lines 246-249.

2. The authors contend that a RANKL antibody control group is unnecessary based on some previous studies that did not use the appropriate controls. Most of the papers presented in proof, are very dated and/or published in low impact journals and/or were simply poorly reviewed. The important of complete controls, cannot be underemphasized. In this case it is not possible to conclude that increased RANKL is driving the events simply because depleting RANKL ablated the effect. Suppressing osteoclasts by any means (as previously explained) would reduce baseline resorption and prevent bone loss. This is not prima facia evidence of a cause-effect relationship, and consequently, the authors should not over-interpret the data and claim that this experiment proves RANKL involvement. While increased RANKL is likely, all that can be concluded from this experiment is that an anti-resorptive strategy may also prevent bone disease in human patients.

As suggested by the reviewer, we have revised the manuscript to interpret the data more appropriately.

In section of Abstract, we replaced “The blockade of RANKL by the anti-RANKL antibody could prevent bone loss, indicating that the increased RANKL contributes to the pathological bone resorption in the context of schistosome infection.” with “The blockade of RANKL by the anti-RANKL antibody could prevent bone loss in the context of schistosome infection.” in our revised manuscript on page 2 lines 30-32.

In section of Introduction, “Here, we found that RANKL promoted schistosome-induced bone loss” was revised to “Here, we found that the increased RANKL was associated with schistosome infection-induced bone loss” in our revised manuscript on page 5 lines 77-78.

In section of Results, “RANKL promotes schistosome infection-induced bone loss *in vivo*” was revised to “The increased RANKL is associated with schistosome infection-induced bone loss *in vivo*” in our revised manuscript on page 7 line 112.

In section of Discussion, we replaced “In conclusion, we demonstrated that RANKL exacerbated the host’s osteoclast-mediated bone loss in the context of schistosome infection” with “In conclusion, we demonstrated that the increased RANKL was correlated with the host’s osteoclast-mediated bone loss in the context of schistosome infection” in our revised manuscript on page 14 lines 246-247.

3. uCT Indices. I was not suggesting that you report all of the indices in the Guidelines, only that the authors use the recommended units and document the methodology as indicated. These guidelines were developed and adopted by the American Society for Bone and Mineral Research, so that different studies can be compared directly and parameters reported without confusion. For example, “bone volume over tissue volume (Tb. Vol/TV)” is neither the correct index nor notation. This should be bone volume fraction (BV/TV). Cortical bone volume as a fraction of total bone volume (Co. Vol/TV) Co. is also not the appropriate unit. This should be Cortical area fraction and is abbreviated as Ct.Ar/Tt.Ar. The guidelines state: “The minimal set of variables that should be reported for cortical regions includes total cross-sectional area (Tt.Ar), cortical bone area (Ct.Ar), cortical thickness (Ct.Th), and cortical bone fraction (Ct.Ar/Tt.A)”

As suggested by the reviewer, we have added the recommended indices with correct notation including tissue volume (TV), bone volume (BV), bone volume fraction (BV/TV), cortical bone thickness (Ct.Th), total cross-sectional area (Tt.Ar), cortical bone area (Ct.Ar), and cortical bone fraction (Ct.Ar/Tt.Ar) in new Figures 1D, 1E, 3G, 5I, S4C, and S5C in our revised manuscript.

4. The non-standard indices generated by your Brucker are very confusing and should be converted to common notation used in the Guidelines. There are many Skyscan users who report data in the expected format so it may necessary to request help from Brucker in converting the indices or units.

As suggested by the reviewer, we have replaced the non-standard indices with the recommended indices and units in our revised manuscript.

5. Furthermore, none of the machine settings are presented (e.g. integration time, tube voltage and current), number of slices and where they were measured. Please upgrade the methods to normal bone specifications.

As suggested by the reviewer, we have added the machine settings and region of interest selection for trabecular and cortical bone in the section of Materials and Methods in our revised manuscript as follows:

The parameters of the scan in the 1176 μCT scanner are as follows: Scan duration (6-7 minutes), X-ray voltage (50 kV), X-ray current (455 μA), number of slices (329), Filter (0.5 mm aluminium), Image pixel size (17.76 μm), Camera pixel size (12.59μm), Rotation step (0.6), and Frame averaging (1).

Both trabecular (metaphyseal) and cortical (metaphyseal-diaphyseal) were selected with reference to the growth plate. The trabecular region commenced about 0.3552 mm (20 image slices) from the growth plate level in the direction of the metaphysis, and extended from this position for a further 1.4208 mm (100 image slices). The cortical region commenced about 2.4864 mm (140 image slices) from the growth plate level in the direction of the metaphysis, and extended from this position for a further 0.5328 mm (170 image slices).

6. Some new uCt has been added for the RANKL ablation experiment Figure 3G, and in 5I but trabecular BMD presented is again not the key index and BV/TV needs to be shown here instead. The data in the supplemental section for BV/TV looks quite good so no reason not to present this as the primary data. The BMDs presented is a dispensable index in uCT.

As suggested by the reviewer, we have added the BV/TV in new Figure 3G and Figure 5I, and moved BMD into supplementary figures in our revised manuscript.

Reviewer #3: (No Response)
